# Exploring a root cause analysis team's experience with involving patients and next of kin after a sentinel event

**DOI:** 10.3389/frhs.2026.1825372

**Published:** 2026-07-17

**Authors:** Silje Liepelt, Hildegunn Sundal, Ralf Kirchhoff

**Affiliations:** 1 Department of Health Sciences, Ålesund, Faculty of Medicine and Health Sciences, Norwegian University of Science and Technology, Ålesund, Norway; 2 Faculty of Health Service and Social Care, Molde University College, Molde, Norway

**Keywords:** healthcare quality, next-of-kin, organisational learning (OL), patient involvement, patient safety, quality research, root cause analysis, sentinel events

## Abstract

**Background:**

There is a growing recognition among healthcare providers and policymakers of the importance of involving patients and their next of kin in root cause analysis (RCA) to improve patient safety and healthcare quality. However, such involvement is intricate and challenging. This study explored how the involvement of patients and next of kin influenced the RCA team's understanding of a fatal sentinel event in maternity care.

**Method:**

The study was a qualitative exploratory single-case study combining document analysis with interviews with six RCA team members. The selected case came from a Norwegian hospital responding to a sentinel event involving the unexpected death of a child during childbirth. The study addressed the following research questions: What were the team's experiences of involving patients and next of kin in the RCA process? Data collection included twelve interviews with six RCA team members, which were analysed using reflexive thematic analysis.

**Results:**

Four main themes were identified. These concerned: (1) uncertainty regarding whether bereaved parents should be involved in the RCA process; (2) challenges associated with conducting interviews with the parents; (3) incorporation of patient and next-of-kin perspectives into the RCA report; and (4) the RCA team's learning process following involvement of the parents.

**Conclusion:**

The findings suggest that involving patients and next of kin in RCA processes may contribute perspectives that strengthen organisational learning and understanding of sentinel events. Future research should explore how RCA frameworks can be systematically adapted to accommodate the involvement of bereaved relatives across different healthcare contexts.

## 1 Introduction

According to the 2019 World Health Organization (WHO), adverse events resulting from unsafe patient care rank among the top ten causes of death and disability globally (1). Adverse events occur in nearly 10% of hospital admissions, and of these, 40%–50% could have been prevented (2–6). In Norway, adverse events occur in 7%–8% of all hospital admissions (7), affecting approximately one in ten patients (8). Such events often arise from complex interactions between clinical, organisational, and systemic factors.

Patients and their family members, often called “next of kin,” bear the most significant impact when a sentinel event occurs in healthcare services (9). Sentinel events are patient safety incidents that have or could have resulted in harm or death (10), incidents wholly or partially caused by poor quality of care (11, 12). Sentinel events require urgent investigation and response to prevent their recurrence (11). The importance of learning from serious adverse events has been highlighted internationally since the publication of *To Err Is Human* (13), which drew attention to the substantial burden of preventable harm in healthcare. Involving patients and families in the post-event analysis is both a moral obligation and a valuable source of information for improving healthcare systems (14). Vincent et al. (15) argue for significant changes in the approach to analysing such events, advocating for patients and families to be partners in the investigation and engaging them from the beginning where possible.

Root cause analysis (RCA) is widely used to identify the underlying causes of sentinel or adverse events and develop corrective actions (10). However, RCA has also been criticised for relying on overly linear understandings of causation and for insufficiently addressing the complexity of healthcare systems (16). Patients and their next of kin are often inconsistently involved in the RCA process, and limited guidance is available on best practices (17, 18).

Previous research suggests that patients and families often hold perspectives on adverse events that differ from those of healthcare professionals and investigators (19). Involving patients and relatives in safety investigations may therefore contribute to organisational learning, increased transparency, improved trust, and more comprehensive understandings of adverse events (18, 20, 21). Nevertheless, relatively little research has explored how patient and next-of-kin involvement influences RCA processes in practice and how RCA teams experience such involvement following sentinel events.

In this study, patient and next-of-kin involvement refers specifically to involving patients and bereaved relatives as informants in the RCA process through interviews and dialogue concerning their experiences and perspectives related to the sentinel event. The study does not examine patient participation as co-investigators or decision-makers within the RCA process.

The Norwegian RCA guidelines increasingly emphasise openness, information sharing, and involvement of patients and relatives following serious adverse events (22). Healthcare organisations are encouraged to establish procedures for involving patients and next of kin during investigation and follow-up processes. However, practical implementation remains challenging, and healthcare organisations may lack established routines and experience regarding how bereaved relatives should be involved within RCA processes.

While open disclosure of sentinel events is crucial for transparency and building trust between healthcare providers (18), healthcare institutions often fall short of meeting the needs and expectations of patients and their families. They increasingly seek more substantial involvement and greater transparency and accountability throughout the process, including post-event analysis and subsequent improvement measures. Experience from other fields suggests that meaningful and influential patient involvement in patient safety requires significant effort (23). Involving patients and their relatives following a sentinel event can be challenging for the hospital, the affected healthcare workers, and the RCA team, as they frequently struggle to meet the needs and expectations of those involved.

This article builds on a previously published study exploring the RCA team's broader experiences of conducting an RCA following the same sentinel event (20). Whereas the previous study focused on organisational and professional experiences of the RCA process overall, the present study specifically re-analyses the empirical material to explore how patient and next-of-kin involvement influenced the RCA process and the team's understanding of the sentinel event.

This study aims to deepen our understanding of the effective involvement of patients and next of kin in an RCA process and the practical challenges an RCA team may encounter. Based on a sentinel event in a Norwegian hospital in 2021 in which a child died unexpectedly during childbirth, this research explores from the perspective of the RCA team how the patient and next of kin influenced the RCA process. The research question is: *What were the team's experiences of involving patients and next of kin in the RCA process?* In this context, we are keen to explore whether the contribution of the patient and next of kin in the chosen single case changed the understanding of the sentinel event and the chronological RCA process. We hope our research and the case in this article add to existing knowledge about RCA processes involving patients and their families.

The following section presents the background, recommendations, and justifications for patient and next-of-kin participation in the analysis of sentinel events, describes the research design and data, and explains the case-study method used to explore the research question.

## 2 Study context

### 2.1 Background

Healthcare providers increasingly recognise the importance of involving patients and their families in incident investigation processes following serious adverse events (19, 24, 25). Patients and next of kin may contribute important experiential knowledge regarding care trajectories, communication processes, time sequences, and the consequences of adverse events that are not always fully visible within clinical documentation alone (19, 21, 23). Previous research suggests that involving patients and relatives in safety investigations may strengthen organisational learning, transparency, and trust-building following sentinel events (18, 23). Interviews with patients and relatives may further provide first-hand accounts that complement healthcare professionals' perspectives and contribute to more comprehensive understandings of adverse events (19, 24). Nevertheless, practical guidance regarding how patients and relatives should be involved in RCA processes remains limited (17, 18).

### 2.2 Regulation of patient and next-of-kin participation in RCA within the Norwegian context

Countries vary considerably in how patients and next of kin are involved following sentinel events and in how such involvement is regulated (26). Norwegian healthcare legislation and national guidelines increasingly emphasise openness, information sharing, and involvement of patients and relatives following serious adverse events (22, 27, 28). These expectations are anchored in both healthcare legislation and regulatory frameworks governing specialist healthcare services (28, 29). Hospitals are expected to communicate with affected patients and next of kin, offer follow-up meetings, and investigate sentinel events to support organisational learning and patient safety improvement. This includes obligations related to information sharing following injury or serious complications (30).

The Norwegian RCA guidelines further recommend involving patients and relatives during investigation processes (22). However, healthcare organisations may vary considerably in how such involvement is operationalised in practice, and established procedures for involving bereaved relatives in RCA processes remain limited.

### 2.3 Root cause analysis in patient safety

Root cause analysis (RCA) is a structured investigation method commonly used following serious adverse events in healthcare to identify contributing factors and support organisational learning (1, 10). RCA processes typically involve interviews, document reviews, and interdisciplinary analysis of the event trajectory. Effective responses to adverse events require both systematic investigation and organisational learning processes that extend beyond identification of immediate causes (31).

Although RCA remains widely used internationally, questions have been raised regarding its effectiveness in preventing recurrence of adverse events and its ability to address the complexity of healthcare systems (32, 33). RCA continues to be recommended internationally as a structured approach for investigating patient safety events and identifying opportunities for system improvement (34). Furthermore, the extent to which patients and next of kin are meaningfully involved in RCA processes varies considerably, and evidence-based guidance regarding effective involvement strategies remains limited (16, 17). Previous research suggests that patients and relatives may contribute perspectives that complement professional accounts and clinical documentation (17, 19). However, there remains limited empirical knowledge regarding how RCA teams experience involving bereaved relatives in practice.

## 3 Materials and methods

### 3.1 Study design

This study employed an exploratory single-case design informed by the methodological work of Schwandt and Gates (35), Yin (36, 37), Simons (38) and Creswell (39) to examine RCA team members' experiences of involving patients and next of kin following a sentinel event. The findings from the team's experience of the RCA process as a whole are published in a previous article by Liepelt et al. (20). The present article represents a focused re-analysis of the same empirical case, specifically examining how patient and next-of-kin involvement influenced the RCA process and the team's understanding of the sentinel event. While the previous publication explored the RCA process more broadly (20), this analysis concentrated specifically on experiences, interpretations, and learning processes related to bereaved relatives' involvement. The interview material was therefore re-analysed with a distinct analytical focus on patient and family involvement.

The case was bounded by time, activity, and organisational context. Detailed information was collected through interviews and document review throughout the RCA process. Table 1 outlines the involvement of patients and next of kin in the selected case and defines the boundaries of the case study.

**Table 1:** Patient and next-of-kin involvement in this case study.

Aspect	Details
Study approach	Exploratory descriptive single-case study
Data collection	Individual in-depth interviews with all RCA team participants before and after the RCA process
Documents analysed	(1) Interviews with RCA team members; (2) written mandate from the process owner; (3) clinic procedures; (4) final RCA report; (5) healthcare legislation and other legal texts; (6) “Risk and Incident Analysis”—Norwegian Handbook for the Health Service
Data analysis method	Qualitative thematic analysis and document analysis
Purpose of interviews	To understand the team members' experiences of patient and next-of-kin involvement in the RCA process
Information extracted	(1) Method of involvement; (2) Purpose of involvement; (3) Who was involved (4) Lessons learned
Participant characteristics	Interdisciplinary RCA team members, including medical specialists, quality advisors, Chief Quality Officer (CQO), and other relevant healthcare professionals
Research design	Exploratory case study focused on understanding patient and next-of-kin involvement in the RCA process in Norway

The Norwegian national RCA guidelines (22) informed the development of the interview guide. Follow-up interviews were conducted to explore participants' experiences across different phases of the RCA process and to allow reflection on issues emerging from the initial interviews (40). Before conducting the second round of interviews, the first author transcribed and conducted a preliminary thematic analysis of the first interviews to identify topics requiring further exploration.

Interview data and relevant organisational and regulatory documents were collected and analysed throughout the research process (Table 2). The first interviews focused on participants' experiences prior to and during the early stages of the RCA process, whereas the second interviews explored experiences related to the later phases of the investigation and final report. The analysis specifically explored how patients and next of kin were involved, how their contributions were interpreted by the RCA team, and whether their involvement influenced the team's understanding of the sentinel event. The study was reported in accordance with the Consolidated Criteria for Reporting Qualitative Research (COREQ) checklist (41).

**Table 2:** Overview of data and analysis method used in the study.

Data sources	Analysis method	Description
Individual in-depth interviews with the RCA team	Qualitative thematic analysis	Sequential interviews conducted with all RCA team participants before and after implementation, focusing on their experiences in the RCA process
Written mandate from the process owner	Document analysis	Analysis of the written mandate from the process owner, outlining the clinic manager's desired answers and goals for implementing the RCA process
Clinic procedures	Document analysis	Examination of clinic procedures used by the RCA team during implementation, comparing previous approaches with new methods adopted in connection with the RCA process
Final RCA report	Document analysis	Analysis of the unofficial report summarising the RCA process, collaboratively written by the CQO and other team members, delivered to the process owner
Healthcare legislation and legal texts	Document analysis	Examination of excerpts from legal texts describing the duties of healthcare organisations and personnel related to the case
“Risk and Incident Analysis”—Handbook for the Health Service	Document analysis	Analysis of the national supervisors’ handbook, which provides information on the health service's RCA methodologies

### 3.2 Setting and sample

Purposive sampling was used to identify a case relevant to the research question, specifically an RCA team involved in the investigation of a sentinel event. Following recruitment of Norwegian hospitals with experience using national RCA guidelines, one hospital agreed to participate in the study during preparation for an RCA process after a sentinel event.

The study was conducted at a medium-sized Norwegian hospital with approximately 6,000 employees, where RCA had been implemented in 2016 and was conducted approximately 2–4 times annually. The study focused on healthcare professionals participating in the RCA team throughout the investigation process (Table 3).

**Table 3:** Characteristics of participants included in the interdisciplinary RCA team.

Participants	Position in healthcare organization	Role in RCA team	Profession	Background information relevant to the RCA process
Medical specialist manager	Safety and Quality Department medical advisor, a senior position in the hospital trust. Directly managed by the subject director.	Team Facilitator. The team facilitator played a vital role in overseeing the methodology during the implementation of the RCA, with a specific emphasis on maintaining the RCA process at the system level.	Physician	Involved in all RCAs in the organisation (approx. 15). Has worked there since 2015 and completed a national method course in RCA at the Directorate of Health in Oslo in 2015. Overall responsibility for patient complaint cases.
Quality advisor/Coordinator in the clinic	Safety and Quality Department	Team Facilitator. Emphasising the methodology during the implementation of the RCA was a key aspect, with additional attention given to supporting the RCA team leader.	Nurse (RN)	Twenty years of neonatal intensive care experience. Two years of experience as a quality advisor/coordinator, including participating in 2 RCAs. Completed national method course in RCA at the Directorate of Health in Oslo in 2019.
Chief quality officer (CQO)	Safety and Quality Department. Receives all orders for RCAs in the hospital trust.	RCA Team Leader	Nurse anaesthetist, educated in management	Quality manager since 2010. Responsible for the safety program in the organisation. Completed national method course in RCA at the Directorate of Health in Oslo in 2016. Introduced RCAs in the organisation in 2016. Since 2016, the organisation has conducted 2–4 RCAs per year.
Senior physician in anaesthesiology	Head of Anaesthesiology	Team Member, Subject Matter Expert, External “In-House” Medical Expert. Physician leader from another hospital in the same organisation. Shares a similar discipline and possesses comparable process knowledge to the second victim in this case.	Physician, specialist in anaesthesiology	Ten years of anaesthesiology experience. No previous RCA experience. No formal training in RCA methodology.
Gynaecologist/Obstetrician	Senior Physician in Obstetrics	Team Member, Subject Matter Expert, Internal Medical Expert	A medical doctor (MD) with a specialisation in obstetrics and gynaecology	Ph.D. in obstetrics (2020). No formal training in RCA methodology.
Senior paediatrician	Senior Physician in Paediatrics	Team Member, Subject Matter Expert, Internal Medical Expert	Physician, specialist in paediatrics	Senior physician since 2017. Assistant physician from 2012 to 2016. Previous experience in conducting an RCA process. No formal training in RCA methodology.

The interdisciplinary RCA team consisted of six members: three representatives from the quality management department [Chief Quality Officer (CQO), quality adviser, and specialist medical manager] and three physicians with clinical expertise relevant to the case, hereafter referred to as “medical experts.” The first author conducted interviews with all participants. To preserve participant confidentiality, quotations are attributed to either RCA facilitators (members of the quality and patient safety department) or medical experts (clinical members of the RCA team). Quotations are further identified according to whether they originated from the pre-RCA or post-RCA interviews. We did not assign individual identifiers (e.g., RCA Facilitator 1 or Medical Expert 2) because the RCA team consisted of only six participants investigating a single, highly specific and publicly known sentinel event. In combination with the participant characteristics presented in Table 3, further differentiation of quotations could increase the risk of deductive disclosure and participant identification.

### 3.3 Data collection

In January 2021, the first author contacted Norwegian hospitals to identify RCA teams experienced in applying the national RCA guidelines. Four hospitals were initially recruited, and one hospital agreed to participate following a sentinel event within the organisation.

Data collection was conducted from May to August 2021 and included twelve individual interviews with six participants (Table 3). Each participant was interviewed twice: once during the early stages of the RCA process and once after completion of the final report. Due to COVID-19 restrictions, all interviews were conducted digitally. Interviews lasted approximately 1.5 h, were audio-recorded, and subsequently transcribed verbatim. All interviews were conducted by the first author.

### 3.4 Thematic coding processes

To analyse the interviews, we employed reflexive thematic analysis (42). The analytic process unfolded through six phases: (1) dataset familiarisation; (2) data coding; (3) initial theme generation; (4) theme development and review; (5) refining, defining, and naming the theme; and (6) writing up.

The initial step (1) of the inductive thematic analysis required the first author to transcribe the interviews, with the entire research team actively reading and re-reading the data material to uncover meanings and patterns. An inductive, data-driven approach was applied to identify themes strongly connected to the data without fitting them into pre-existing coding frames or analytic preconceptions (43).

In the second phase (2), coding was conducted independently by both the first and last author (see Table 4). Initial codes were subsequently reviewed and compared to explicitly capture ideas relevant to our research questions. We concentrated on systematically interpreting semantic approaches to capture specific and complex concepts and explicit meanings pertinent to our research questions (participant-driven). NVivo version 20/1,3 (for Windows) was utilised for data analysis.

**Table 4:** Example of the coding process in the interview dataset.

Data extract	Code	Subthemes	Theme
“This time, we chose to interview the parents. As far as I understood, they had not spoken to relatives before, or maybe they had done it once in a previous analysis, but we did not do it in the two analyses I was involved in last time. The reason for this was that in the last analysis, the one I had before this one last year, when this was discussed with the parents, they asked why *they* hadn't been talked to. Because they hadn't been able to share their version. And then I thought we should take that into account for another time. So, this time, I brought it up and said, ‘Should we talk to the parents this time? We need to remember the feedback we received last time!’” “I got a bit of a response like, no, they didn't have a tradition for that. So, I asked the quality manager to check what the method says. The method does mention that one should talk to relatives and offer them the opportunity to tell their version. Yes, and then he was a bit like, “We can check with the clinic manager!” And she just dismissed it, saying, “No, it was not necessary to talk to the parent”. And it's probably a bit like what we thought last time that they have had a trauma, they have experienced losing a child—so if we come and ask, you think that would be wrong. It's easy to think that! That it's out of concern for them! But I felt that I was still a bit insistent and thought that it depends on who these parents are! Whether they are the type to be asked!”	Patient/next-of-kin involvement (shift of meaning from the same informant) Method/guideline checking Clinical manager involved	Scepticism about involvement Lack of internal procedures Involvement of the clinic manager in decision-making Quality Advisor role—acts as “ombudsman”	The RCA team's assessments prior to the interview with the parents

In the third phase (3), shared patterns of meaning across the dataset were identified, and the research team constructed subthemes and themes (see Table 5). In the fourth phase (4), themes were reviewed to enhance the understanding and identification of underlying patterns of meaning. The fifth phase (5) involved creating a brief synopsis for each theme. Lastly, in the sixth phase (6), we wove together our analytic narrative to produce the introduction, methodology, and conclusion sections. In employing this thematic coding process, we aimed to unlock nuanced insights embedded in our interviews, contributing depth and clarity to our understanding of the research question. The themes in Table 4 are presented chronologically with respect to the unfolding of the RCA process in the selected case. We have summarised our most central findings, which show the main themes and sub-themes that emerged in our analysis. Furthermore, we have assessed the case and what the processes of the various phases yielded (see Table 5: Main themes, subthemes, and outcomes of the chronological RCA process). Reflexivity was considered throughout the research process. The authors have prior professional experience related to patient safety, quality improvement, and RCA processes within Norwegian healthcare settings. These experiences informed the interpretation of the data while also requiring continuous reflection regarding potential preconceptions and analytical assumptions. Themes and interpretations were therefore discussed continuously within the research team to strengthen analytical rigour and challenge individual interpretations.

**Table 5:** Main themes, subthemes, and outcomes of the chronological RCA process.

Main themes	Subthemes	Outcomes of the chronological RCA process
RCA team's assessments prior to the interview with the parents	Scepticism about involving parents Lack of internal procedures Clinic manager involved in decision-making Quality Advisor's role—acts as “ombudsman”	The decision to involve patient/next of kin
The interview with the parents	Parents' reactions and strong emotions The mother's breathing difficulties during the sentinel event not sufficiently observed and documented Handling the interview situation	RCA team's discomfort
Patient and next-of-kin voice in the RCA report	Careful formulations to respect all audiences for the report The report presented to the parents at their home	Considerable attention to wording and tone in the final report
The learning process undergone by the RCA team as a result of involving the parents	Rebuilding trust among patient and next of kin and healthcare personnel Sharing results and experiences The mother's perspective provided useful subjective information	Trust and learning

### 3.5 Ethical considerations

The Regional Committees for Medical and Health Research Ethics (REK) (reference #195549) determined that the project, falling under the scope of a quality improvement initiative rather than medical and health research per the Health Research Act, could proceed and be published without REK approval. The project adhered to data privacy guidelines, with no collection of personally identifiable data beyond the RCA team members. The Norwegian Centre for Research Data (NSD) (project number 562024) supervised the handling of personal information, including complying with relevant laws and General Data Protection Regulation (GDPR). Informed consent was obtained from all participants. The initiative, recognised as a quality improvement project, empowered the hospital to make decisions regarding essential patient-related information by referring to the Norwegian Act relating to health personnel (Sections 26 and 29c). Participants shared sensitive information about themselves and third parties. In response, the research team developed strategies for context-sensitive approaches to honour the richness of the interview material while ensuring participant protection. Permission from the CQO was obtained to address potential ethical concerns in publishing research data. Internal documents used in the RCA analysis were exempt from public access. To navigate the delicate balance between two conflicting priorities—maximising the protection of participants' identities while preserving the value and integrity of the data (44)—quotations were anonymised through the use of broad role descriptors and interview phase identifiers rather than specific professional titles. Individual participant identifiers were deliberately omitted because the small sample size and the publicly known nature of the case could have enabled identification of participants through deductive disclosure. Furthermore, access to unofficial internal documents was restricted.

## 4 Results

The themes identified through the analysis are summarised in Table 5.

The results are presented in two parts. First, a brief description of the sentinel event is provided based on the final RCA report. Second, four themes related to patient and next-of-kin involvement in the RCA process are presented:

1. The RCA team's assessments prior to interviewing the parents,
2. The interview with the parents,
3. Patient and next-of-kin voice in the RCA report, and
4. The RCA team's learning process following involvement of the parents

### 4.1 Description of the sentinel event (the RCA team's summary from the final report)

A woman giving birth [number of births], identified as a high-risk birth due to known gestational diabetes and a previous caesarean section, is admitted for induction of labour at week [weeks of pregnancy]. She receives a birth epidural assessed to have little effect. A new epidural is placed, after which the woman gets high spinal/blockage symptoms. The anaesthesia takes over with subsequent paralysis of the muscles, leading to breathing difficulties and signs of hypoxia. An emergency caesarean alarm is triggered, and the baby is delivered quickly but emerges pale and lifeless. Life-saving treatment is carried out on the baby. The baby's heart is successfully started; however, the child has symptoms of extensive damage compatible with oxygen deprivation. The treatment ends the next day. The baby is declared dead around 12 h after birth.

### 4.2 Themes from the analysis

#### 4.2.1 The RCA team's assessments prior to the interview with the parents

The RCA team initially expressed uncertainty regarding whether and how the parents should be involved in the RCA process. Although informing patients and relatives about ongoing investigations was standard practice, interviewing bereaved parents as part of the RCA was not routinely established within the organisation.

Several participants described internal organisational disagreement regarding whether bereaved parents should be directly involved in the RCA process. Communication between the parents and the analysis team was coordinated by the Chief Quality Officer (CQO). Initially, the CQO opposed interviewing the parents, partly because there was no established tradition for doing so and partly because the clinical manager/process owner disagreed with including them.

One participant explained: “We had no established tradition for involving relatives directly in the RCA interviews” (RCA facilitator, pre-RCA interview).

Participants also highlighted uncertainty regarding the mother's dual role as both patient and bereaved parent. Interviewing the mother required the team to address both her experiences as a patient and the parents' experiences following the loss of their child.

The quality adviser played a key role in advocating for involvement of both parents and referred to national guidelines recommending inclusion of next of kin in RCA processes. According to the quality adviser, some of the initial scepticism reflected limited organisational experience and lack of training in communicating with bereaved relatives after a patient death.

One participant reflected: “We were not really trained to have these kinds of conversations with parents after losing a child” (RCA facilitator, pre-RCA interview).

Participants further described that previous experiences within the organisation had contributed to awareness regarding potential exclusion of relatives from investigation processes. Reflecting on a previous RCA, the quality advisor recalled that the parents had questioned their execution from the process: “They asked why they had not been interviewed in the process and thought they had a lot to contribute to the process” (RCA facilitator, post-RCA interview). After internal discussions and consulting with the professional community, the RCA team decided to interview both parents.

#### 4.2.2 The interview with the parents

During the interview, the parents emphasised the significance of sharing their experiences and insights concerning the fatal sentinel event, especially regarding the loss of their child. The parents, particularly the mother, were willing to engage with the analysis team and share details from the event with the team members. The mother had previously expressed to the professional community her frustration and anger, which the analysis team considered a natural response after losing a child. This led to a discussion within the analysis team about who should conduct the interviews. The quality advisor, drawing on her extensive experience in the neonatal intensive care unit and her training in communicating with bereaved parents, felt confident in taking on this task. She considered follow-up interviews with parents who have lost a child to be an important part of her work despite the inherent challenges. She was also trained to conduct conversations with relatives in crisis. The informant emphasized the importance of nurses' communication skills when supporting bereaved parents. She described nurses as highly skilled communicators who frequently spend substantial time with parents during difficult circumstances: “There is a great deal of difficult communication involved in these situations, and the nurses here are very skilled at communicating with parents during these challenging circumstances. They are often the ones who spend the most time alongside the parents” (RCA facilitator, pre-RCA interview).

It was deemed essential to provide relatives the opportunity to share their perspectives on the case. After a discussion, the analysis team agreed to have three members present at the interview with the parents to ensure comprehensive support and engagement. They acknowledged the importance of having multiple team members present during the interview, which would allow them to be responsible and to “address both parents” effectively, as both were providing information as informants. “There were two of them, and both were informants, so we needed to be able to address both parents” (RCA facilitator, post-RCA interview).

The team conducting this interview described it as a challenging conversation. The interviewer noted that the mother initially directed aggression toward the analysis team. One participant described that the mother initially expressed considerable anger and frustration towards the analysis team: “She was quite angry in the beginning” (RCA facilitator, post-RCA interview). However, the dynamics evolved, as the team assured the parents that they did not need to explain or defend the employees' actions. Subsequently, the mother continued to share her version of the incident with the analysis team.

During the conversation, there were moments of silence, which the presence of multiple team members helped address. The quality advisor found conducting the interview challenging, emphasising the importance of absorbing information without constantly planning the next question: “There is a pulse in interviewing, being able to sit and take in what people are saying without thinking about what you will ask next” (RCA facilitator, pre-RCA interview). The team recognised the value of having two interviewers to share the responsibility, relieving the pressure on each. Participants described that managing silence and emotional pauses became an important relational aspect of the interview process. Several team members reflected on the importance of allowing space for emotional reactions while simultaneously maintaining the structure of the investigation interview.

During the interview, the mother recounted her experience of not being able to breathe during the labour and described it as a prolonged struggle. The analysis team noted that the healthcare personnel responded swiftly and professionally, while the mother took a subjective approach to the incident. The mother stated during the interview that she had experienced “blackouts” in connection with the second epidural and that she could not breathe for an extended period before she received respiratory support. She recalled that “now and then it went black for her.” The healthcare personnel involved had not documented these observations, and the analysis team concluded that this could be due to the mother falling in and out of consciousness. It was pointed out that it was solid and vital to hear the parents' description of the incident. “They had many things to tell, and they needed to be allowed to tell their story” (RCA facilitator, post-RCA interview).

The situation became particularly complex because the mother and the health personnel had differing accounts of the event. In response, the analysis team emphasised that they had nine different informants, including the parents, and tried to contextualise these perspectives within different timelines and narratives. The mother's experience of not being able to breathe became a focal point. “It was a vital observation that was not documented in the patient record” (Medical expert, post-RCA interview).

#### 4.2.3 Patient and next-of-kin voice in the RCA report

As the final report was being written, the complexity of the task became apparent, particularly the importance of carefully selecting language and tone. The goal was twofold: to support the parents' grieving process and simultaneously serve as an effective communication tool for healthcare personnel. The interview with the parents revealed certain aspects of the incident that the medical experts found inadequately documented in the medical record. One participant reflected that the mother's description of breathing difficulties constituted “powerful reading” and contributed important information that was absent from the clinical documentation. “It indicates that she was short of breath for a while, so it also gives a bit on the professional level – but first and foremost, it is powerful reading!” (Medical expert, post-RCA interview). This reading increased the motivation of medical experts to conduct a thorough RCA and encouraged healthcare personnel to learn from the incident and prevent its repetition.

While waiting for the final report, the parents actively requested the result of the analysis work to move forward. Despite the clinical manager bearing formal responsibility for follow-up with the patient and next of kin, the analysis leader took responsibility for this task. During the process, the mother frequently exchanged text messages and had multiple phone conversations with the analysis leader because the parents eagerly awaited the final report. The interaction with the mother and the healthcare personnel involved made it clear that they did not want to postpone anything unnecessarily and felt obligated to avoid delays. These perspectives influenced the analysis leader, who strived to be a “time optimist.” The analysis team found satisfaction in the perspectives provided by the parents, adding a new dimension to their understanding of the sentinel event. This input offered a unique subjective insight, especially concerning the mother's temporal perception of the event and complemented the objective information obtained from medical records and healthcare professionals.

It was emphasised that while the interview with the parents did not uncover significant new information, it provided additional insight into the mother's experience. The interview shed light on the fact that the mother had severe hypertension. “We gained a greater insight into the experience of being in a situation where you lose control and are exposed to it. And we get a different experience of the consequences of the event.” (RCA facilitator, post-RCA interview). One of the medical experts stated, “It also sheds light on the fact that she has had severe hypertension, although they were unable to measure it.” (Medical expert, post-RCA interview). It was emphasised that her experience of not being able to breathe was a vital observation not documented in the patient's record. The experience of not being able to breathe was seen as a powerful observation both professionally and on a personal level. “It indicates that she was short of breath for a while, so it also gives a bit on the professional level – but first and foremost, it is powerful reading!” (Medical expert, post-RCA interview).

The team acknowledged that this added complexity to the writing process, as they needed to be mindful of how the parents would receive the report. This consideration influenced their choice of how to formulate text passages in the report and the content's overall tone. The final report was designed to facilitate the parents' grieving process while also serving as a communication tool for healthcare personnel. Furthermore, the report provided additional value to the parents as they move forward after the traumatic experience. As one participant reflected, “It brings out the truth of what actually happened” (RCA facilitator, post-RCA interview).

#### 4.2.4 The RCA team's learning process following involvement of the parents

After completing the RCA report, the quality manager, the CQO, and the paediatric ward manager visited the parents in their home to present the final RCA report. During the process, the analysis team gained a deeper understanding of the parents' situation, including how they were coping with the loss of their child and the challenges they faced as immediate family members. Participants described that involving the parents contributed to a deeper understanding of the consequences of the sentinel event and supported the identification of potential areas for improvement within the organisation. Conducting the RCA process was described as an important way of demonstrating that the organisation took the sentinel event seriously for both the patient and the next of kin. “I think it is very important that we do an analysis after such a serious event” (RCA facilitator, pre-RCA interview).

Participants described ongoing disagreement among healthcare personnel regarding the value of the RCA process. Some healthcare professionals questioned whether the investigation represented an unnecessary use of organisational resources because they did not expect the RCA to uncover significant new findings. One noted, “I have not had the opportunity to engage with everyone or stay informed about all the ongoing discussions” (Medical expert, post-RCA interview).

Most healthcare professionals were receptive to being interviewed, although two participants initially expressed reluctance. Several participants later described the interviews as meaningful and partly functioning as a form of reflective debriefing for both healthcare professionals and the parents. “Many have been glad that we did such an analysis and that they got to talk through what happened” (RCA facilitator, pre-RCA interview).

Participants described that the parents expressed appreciation that the healthcare organisation had conducted an RCA process and involved them in the investigation. Several participants perceived that involvement in the RCA reassured the parents that the organisation took the sentinel event seriously and intended to learn from the incident.

Although the parents continued to experience grief following the loss of their child, participants described that the parents valued the opportunity to contribute to the investigation process and prevention of similar incidents. Several participants reflected that involving relatives in RCA processes was important because bereaved families wanted reassurance that the organisation would learn from the incident. “You will get many answers to what you were feeling. It brings out the truth of what happened” (RCA facilitator, post-RCA interview).

## 5 Discussion

This study explored how the involvement of patients and next of kin influenced the RCA process following a sentinel event within a Norwegian hospital context. These findings suggest that experiential knowledge held by bereaved relatives serves as a distinct epistemic resource in RCA processes, one that is not easily substitutable by clinical records or professional accounts. This has implications for how RCA frameworks conceptualise knowledge production: if relatives' perspectives alter how sentinel events are interpreted rather than merely supplementing existing accounts, this challenges models that treat RCA primarily as a technical-analytical process. The findings are particularly relevant within the Norwegian healthcare context, where national guidelines increasingly emphasise openness, patient involvement, and learning following serious adverse events (22, 27, 28). Despite these recommendations, the study demonstrates that uncertainty, limited organisational routines, and differing professional perspectives may complicate the implementation of patient and next-of-kin involvement in RCA processes in practice.

### 5.1 Exploring patient and next-of-kin engagement

Our study contributes to a growing body of research recognising patient and family engagement as an important component of patient safety improvement (45, 46). Although previous research has emphasised the value of involving patients and next of kin following adverse events (27, 47), there remains limited empirical research examining how such involvement influences RCA processes in practice.

Consistent with previous studies demonstrating differing perspectives among patients, relatives, healthcare professionals, and regulatory authorities following adverse events (48, 49), our findings suggest that bereaved relatives contributed experiential knowledge that complemented clinical documentation and professional accounts of the sentinel event. In particular, the parents' descriptions influenced how the RCA team interpreted aspects of the event, reflected on its consequences, and formulated the final RCA report.

The findings further suggest that involving patients and next of kin may influence not only information gathering during RCA processes, but also broader organisational learning processes, including reflection, communication, and perceptions of accountability and transparency within the organisation. Previous research has highlighted that transforming investigation findings into meaningful recommendations remains challenging, suggesting that richer stakeholder perspectives may strengthen learning processes following adverse events (50). These findings support previous studies suggesting that patient and family involvement may strengthen learning processes and patient safety culture within healthcare organisations (18, 51, 52).

While Norway has a relatively limited tradition of involving patients and next of kin in regulatory and investigative processes (21), both international patient safety initiatives and Norwegian governance documents increasingly emphasise patient and family involvement following serious adverse events (18, 51, 52). Our findings illustrate how implementing such involvement in practice may entail both organisational challenges and important learning opportunities for RCA teams.

The following section discusses how Norwegian RCA guidelines influenced the team's assessments and how organisational and emotional factors shaped implementation of patient and next-of-kin involvement in practice.

### 5.2 Guidelines and recommendations

Our findings highlight the potential value of involving patients and next of kin in RCA processes, while also demonstrating the organisational challenges associated with implementing such involvement in practice. Although Norwegian RCA guidelines recommend that patients and relatives should be offered the opportunity to share their perspectives following sentinel events (22), participants in this study described uncertainty and disagreement regarding whether bereaved parents should be directly involved in the RCA process.

The findings suggest that formal recommendations alone may be insufficient to support meaningful involvement of patients and next of kin in RCA processes. Participants described limited organisational routines, lack of previous experience, and uncertainty regarding how to manage emotionally demanding conversations with bereaved parents. These findings indicate that implementation of patient and next-of-kin involvement requires not only procedural guidance, but also organisational preparedness, relational competence, and support structures for healthcare professionals conducting RCA investigations.

The emotional intensity of the interviews with the parents was described as challenging for the RCA team. At the same time, participants experienced that the interviews contributed important experiential perspectives regarding the mother's experience of the sentinel event and influenced how the RCA team interpreted and communicated aspects of the event in the final report. In particular, participants described that involvement of the parents influenced the wording, tone, and communicative considerations embedded in the final RCA report.

Although there was initial disagreement regarding parental involvement, participants ultimately described that involving the parents contributed to broader organisational learning processes and strengthened awareness regarding openness, communication, and inclusion following serious adverse events. Similar learning ambitions have been highlighted in Norwegian supervisory reports examining serious adverse events in hospital settings (53). The findings therefore suggest that involving bereaved relatives may influence not only factual understanding of sentinel events, but also relational and organisational dimensions of RCA processes. Earlier patient safety literature has similarly argued that patient and family perspectives may contribute valuable insights during root cause analyses and subsequent improvement work (54).

### 5.3 Strengths and limitations of the study

A major strength of this study was the use of an exploratory case study design, which enabled an in-depth examination of how patient and next-of-kin involvement influenced the RCA process within a real-world healthcare context. Follow-up interviews conducted across different phases of the RCA process allowed the researchers to capture how participants' experiences and reflections developed over time. Inclusion of organisational and contextual documents further strengthened understanding of the unfolding investigation process.

The study has several limitations that should be acknowledged. First, as a single-case study conducted within one Norwegian hospital, the findings are context-specific and may not be directly transferable to other healthcare settings or RCA processes. Second, the analysis relies exclusively on the accounts of RCA team members, meaning that patient and next-of-kin perspectives are not captured directly but are instead mediated through the interpretations of professionals involved in the process. Although the manuscript discusses how patients and next of kin were involved in the RCA, the analytical lens remains that of the team members, which inevitably shapes what aspects of that involvement are foregrounded. Third, the retrospective nature of the accounts, combined with the emotionally charged circumstances surrounding a sentinel event, may have influenced how participants recalled and described their experiences. Future research should seek to include direct accounts from bereaved relatives to complement and potentially challenge the perspectives reported here.

## 6 Conclusion

This study highlights how involving patients and next of kin in the RCA process influenced the RCA team's understanding of the sentinel event and contributed to organisational learning. Although the RCA team initially expressed uncertainty regarding how to involve bereaved parents, participants ultimately described parental involvement as valuable for understanding the consequences of the event and strengthening the investigation process.

The findings suggest that involvement of patients and next of kin may contribute perspectives not captured in clinical documentation alone and may support trust, learning, and openness following sentinel events. At the same time, the study illustrates the practical and emotional challenges associated with involving bereaved relatives in RCA processes and highlights the need for clearer organisational routines and competence in conducting such involvement.

## Data Availability

The raw data supporting the conclusions of this article will be made available by the authors, without undue reservation.
